# Phase-dependent dynamic potential of magnetically coupled two-degree-of-freedom bistable energy harvester

**DOI:** 10.1038/srep34411

**Published:** 2016-09-28

**Authors:** Pilkee Kim, Minh Sang Nguyen, Ojin Kwon, Young-Jin Kim, Yong-Jin Yoon

**Affiliations:** 1 School of Mechanical and Aerospace Engineering, Nanyang Technological University, Singapore 639798, Singapore; 2 Energy Research Institute @ NTU, Interdisciplinary Graduate School, Nanyang Technological University, Singapore 639798, Singapore

## Abstract

A system of magnetically coupled oscillators has been recently considered as a promising compact structure to integrate multiple bistable energy harvesters (BEHs), but its design is not straightforward owing to its varying potential energy pattern, which has not been understood completely yet. This study introduces the concept of phase-dependent dynamic potential in a magnetically coupled BEH system with two degrees of freedom (DOFs) to explain the underlying principle of the complicated dynamics of the system. Through theoretical simulations and analyses, two distinct dynamic regimes, called the out-of-phase and in-phase mode regimes in this report, are found to exist in the frequency regions of the 1^st^ and 2^nd^ primary intrawell resonances. For the out-of-phase mode regime, the frequency displacement (and output power) responses of the 2-DOF BEH system exhibit typical double-well dynamics, whereas for the in-phase mode regime, only single-well dynamics is observed though the system is statically bistable. These dynamic regimes are also revealed to be caused by the difference in the dynamic potential energy trajectories propagating on a high-dimensional potential energy surface. The present approach to the dynamics of the 2-DOF BEH system can be extended and applied to higher-DOF systems, which sheds light on compact and efficient designs of magnetically coupled BEH chain structures.

A vibration-based energy harvester (VEH) has been developed as an alternative to chemical batteries to power wireless autonomous electronic devices^1^. At the early stages of development, VEHs were designed mostly using a linear electromechanical resonator, which produces a high power only in a narrow resonant frequency band and hence, is not suitable for actual ambient vibration sources with time-varying frequencies or a wide frequency spectrum^2^,^3^. To overcome this limitation, a variety of energy harvesting structures have been investigated, e.g., frequency-tunable oscillators^4^,^5^,^6^, an array of multimodal oscillators^7^,^8^,^9^, stiffness softening (or hardening) oscillators^10^,^11^,^12^,^13^,^14^,^15^,^16^, and bistable (or multistable) oscillators^17^,^18^,^19^,^20^,^21^,^22^,^23^,^24^,^25^,^26^,^27^,^28^,^29^,^30^,^31^,^32^,^33^,^34^,^35^. In particular, during the last decade, a bistable electromechanical oscillator has been considered as one of the most promising systems that efficiently harvests energy from ambient vibrations in a broad frequency band. A bistable energy harvester (BEH) is known to have a double-well potential energy function, and its broadband performance can be achieved by high-energy orbit motion across two potential wells (the so-called interwell motion). However, this potential well escape phenomenon occurs only when the kinetic energy of the system is large enough to surmount the potential barrier between the two wells. For this reason, many theoretical and experimental studies have been devoted to the performance evaluation of BEHs under various types of ambient vibration sources such as swept sine excitations^14^,^19^ and random noises^21^,^22^. On the other hand, a tristable or quadstable energy harvester^27^,^28^ has been proposed as an extended version of a BEH to reduce the potential barriers (thus, the threshold excitation intensity), and several theoretical and experimental studies^27^,^28^,^29^,^30^,^31^,^32^ showed their beneficial effect on the performance improvement over BEHs, especially under low-intensity ambient excitation.

Recently, multiple degree-of-freedom (DOF) BEHs^33^,^34^,^35^ have also drawn attention from researchers that attempted to further enhance the energy harvesting performance in a broader operating frequency bandwidth or under multidirectional vibration sources. Particularly, magnetically coupled multi-DOF BEHs should be potential candidates for compact arrangement of BEH chains, as both bistable structures and noncontact couplings between multiple oscillators can be formed by the proper use of magnetic forces. However, the design of a magnetically coupled multi-DOF system is not straightforward owing to its complicated dynamics. Only in a few reports^33^,^34^,^35^, the potential merits of magnetically coupled 2-DOF BEHs were presented, but deep physical insights into their nonlinear dynamics arising from the varying potential energy pattern with the relative motion of two magnet oscillators were not provided.

This report presents a comprehensible approach to the nonlinear dynamics of the magnetically coupled 2-DOF BEH at the 1^st^ and 2^nd^ primary resonances, introducing the concept of the phase-dependent dynamic potential energy. The 2-DOF BEH comprises two bimorph cantilever beam oscillators with permanent magnets at the free end, as shown in Fig. 1(a). The two tip magnets face each other with same polarities, and accordingly, the two beam oscillators turn to be mutually bistable when the magnetic repulsive force becomes large. In this study, it is assumed for simplicity that the two oscillators on the left and right have the same geometric dimensions and material properties, except for the lengths of the beams. Because two different lengths are used for the two beams (as illustrated in Fig. 1(a)), the 2-DOF BEH system possesses two distinct frequency bands for the 1^st^ and 2^nd^ primary resonances. Thus, this geometric dimension of the 2-DOF BEH is in accordance with the main design objective of multi-modal VEHs to harvest vibration energy in multiple frequency bands. Refer to Table 1 in the Methods section for details of the geometric dimensions and material properties. The potential energy function of the 2-DOF BEH is a function of the relative position of the two oscillators, forming a high-dimensional potential energy surface. This potential energy function is a state function by definition, and thus it should not be misunderstood as a path-dependent function. In fact, only an apparent potential energy path that the 2-DOF BEH experiences during oscillation varies with the excitation intensity and frequency on the prescribed potential energy surface. In this study, such apparent potential energy paths of the 2-DOF BEH system are theoretically examined along with its nonlinear resonant behavior in order to investigate the underlying principles of its complicated dynamics.

## Magneto-electro-mechanical oscillator model

**Figure 1:**
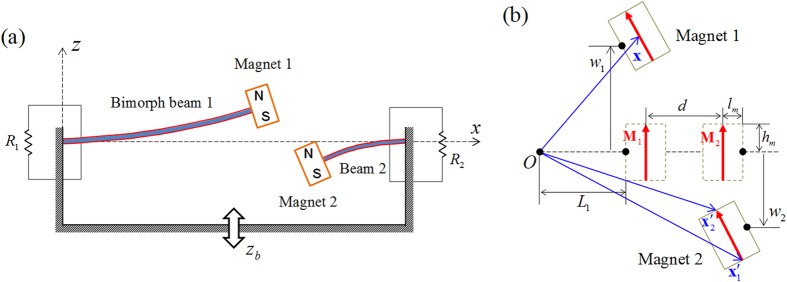
Schematics of (**a**) the magnetically coupled 2-DOF bistable energy harvester and (**b**) geometric configuration of two tip magnets with the coordinate system. In (**a**), the two bimorph cantilever beams on the left and right have the same geometric dimensions, except for their lengths. For each bimorph beam, piezo-polymer layers are fully laminated on the upper and lower surfaces of a metal substrate. These piezo-polymer layers are connected to an external electrical load. Two identical neodymium magnets are used as the tip magnets.

First, a mathematical model of the magnetically coupled 2-DOF BEH is developed. The governing field equations and boundary conditions for two bimorph cantilever beams are derived based on the Euler–Bernoulli beam theory and linear piezoelectricity^25^. Then, the Galerkin projection method is applied to the field differential equations in order to obtain the lumped parameter model. Such a derivation process is somewhat complicated but straightforward, and its detailed steps can be found in many earlier works^25^,^27^. The general form of the magneto-electro-mechanical oscillator model of the 2-DOF BEH can be expressed as


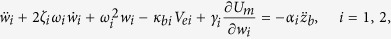



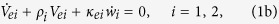


where index *i* is used to denote the quantities for the two beam oscillators on the left (*i* = 1) and right (*i* = 2) in Fig. 1(a); *w*_*i*_ and *V*_*ei*_ are the tip deflection and the output voltage across the electrical resistance, respectively; *ζ*_*i*_ and *ω*_*i*_ are the damping ratio and the natural frequency, respectively; *ρ*_*i*_ is the cut-off frequency; *κ*_*bi*_ and *κ*_*ei*_ are the electromechanical coupling coefficients; 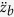 is the base acceleration; and *U*_*m*_ is the interaction energy (or the magnetic potential energy) between two identical permanent magnets by which the two energy harvesting oscillators are coupled.

To derive the analytical expression of *U*_*m*_, the magnetic charge model^29^ is employed for the permanent magnets with the coordinate system given in Fig. 1(b) and, for further simplification, the magnetic poles of each magnet are approximated to the equivalent point charges. Accordingly, for the present system, one magnetic dipole (which corresponds to Magnet 1 in Fig. 1(b)) can be considered to be subjected to an external magnetic field applied by another magnetic dipole (Magnet 2 in Fig. 1(b)). Thus, when the magnets oscillate, the magnetic potential energy that the system acquires is given by


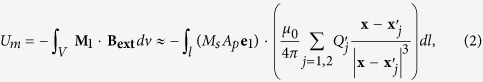


where *μ*_0_ is the permeability of free space; **M**_1_(=*M*_*s*_**e**_1_) is the magnetization vector of Magnet 1; *A*_*p*_ is the cross-sectional area of the magnet or its pole face area; **B**_**ext**_ is the magnetic field generated by Magnet 2; 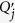 denotes the equivalent magnetic charge of Magnet 2 given by (−1)^*j*^*M*_*s*_*A*_*p*_ at the negative and positive poles (*j* = 1 and 2, respectively); and 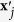 is the position vector of the charge 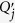. By manipulating Eq. (2) with the geometric configuration of the system presented in Fig. 1(b), the magnetic potential energy can be obtained in the following form:


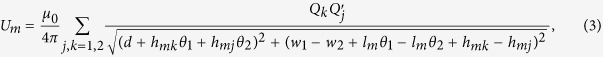


where *Q*_*k*_ = (−1)^*k*^*M*_*s*_*A*_*p*_; *h*_*mj*_ = (−1)^*j*^*h*_*m*_; *h*_*mk*_ = (−1)^*k*^*h*_*m*_; *l*_*m*_ and *h*_*m*_ are the half-length and half-height of the magnets, respectively; and *θ*_*i*_ (*i* = 1, 2) is the rotation angle of the beam at the free end.

The total potential energy of the system model is evaluated by the summation of the strain energy and magnetic potential energy, as follows:


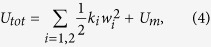


where *k*_*i*_ is the equivalent spring constant of the beam.

## Results and Discussion

### Static bistability

**Figure 2:**
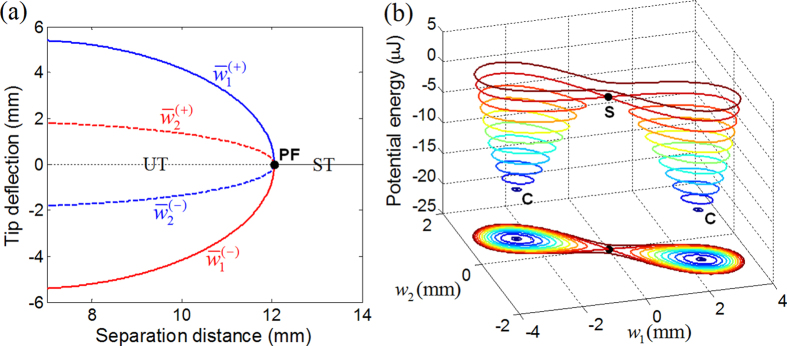
(**a**) Bifurcation diagram of the static equilibrium state with respect to separation distance *d* and (**b**) the contour plot of the potential energy as a function of deflections *w*_1_ and *w*_2_, obtained when *d* = 11.45 mm. In (**a**), the supercritical pitchfork bifurcation of the equilibrium state is denoted by point PF. Through this point, the stable trivial solution (ST) loses its stability and becomes unstable (UT), and concurrently, the two pairs of stable nontrivial solutions, (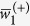, 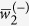) and (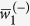, 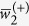), are bifurcated. In (**b**), the total potential energy relative to the trivial state is used for illustration. Points S and C denote the trivial saddle and nontrivial center points, respectively.

The bifurcation analysis is performed on the equilibrium state of the system model (derived through Equations (1)–(3)) in order to investigate its static bistability. For this analysis, separation distance *d* is used as a bifurcation parameter. The variation of equilibrium solution (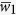, 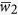) with the decrease in parameter *d* is examined by solving the static homogeneous equations of the system continuously, i.e., 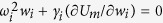 (*i* = 1 and 2). Figure 2(a) shows the bifurcation diagram of the equilibrium state with respect to parameter *d*. A magnetic repulsive force is exerted mutually on the two permanent magnets, and it becomes larger as the separation distance decreases. When the magnetic force reaches the certain intensity level (at point PF in Fig. 2(a)), structural instability occurs in the equilibrium state(s) of the oscillators, accompanying with a pitchfork bifurcation phenomenon. The supercritical pitchfork bifurcation leads to the destabilization of the trivial equilibrium (from ST to UT in Fig. 2(a)) and the generation of the two stable nontrivial equilibria (nonzero branches in Fig. 2(a)), which indicates the transition of the static stability from mono to bistability. Each stable nontrivial equilibrium comprises two components with opposite signs, owing to the magnetic repulsion effect, e.g., a pair of positive 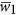 and negative 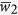 (indicated by 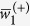 and 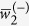, respectively, in Fig. 2(a)) or vice versa (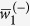 and 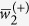 in Fig. 2(a)). The magnitude of static deflection 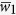 is always larger than that of 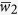 because of a higher compliance of the left beam oscillator. To clearly demonstrate such static bistability, the total potential energy was evaluated as a function of the deflections of the two beam oscillators when *d* = 11.45 mm. Its contour plot is presented in Fig. 2(b). It can be evidently observed that one stationary potential saddle exists at the trivial midpoint between the two nontrivial potential centers; thereby two skew-symmetric potential wells are formed in three-dimensional space.

As discussed above, the magnetically coupled 2-DOF BEH obviously possesses the double-well potential energy function after the supercritical pitchfork bifurcation occurs. The fundamental mechanism of this static bistability seems to be very similar to those of existing 1-DOF bistable oscillators^19^,^28^. However, the actual trajectory of the potential energy that the 2-DOF BEH experiences during its oscillation does not follow a certain fixed path on the three-dimensional potential energy surface in Fig. 2(b), but unpredictably varies with the relative motion of the two magnets, which makes it difficult to design magnetically coupled multi-DOF energy harvesters. To address this problem, we study the nonlinear resonant behavior of the 2-DOF system along with the potential energy trajectory.

### Out-of-phase and in-phase intrawell resonances

All bistable systems exhibit two distinct dynamic motions depending on the intensity of external excitation: small-amplitude intrawell motion (under weak excitation) and large-amplitude interwell motion (under relatively strong excitation). In general, the transition from intrawell to interwell motion (i.e., the potential well escape phenomenon) is likely to occur through intrawell resonances owing to high vibration transmissibility. For this reason, intrawell resonances were considered as the main route to high-energy orbit motion in designing BEHs. For the present 2-DOF BEH, the two primary intrawell resonances exist and the associated vibration modes make differences in the resonant behaviors and potential energy trajectories. The present study mainly focuses on the primary resonant behaviors of the magnetically coupled 2-DOF BEH system and thus do not treat any of the possible secondary or combination resonances.

**Figure 3:**
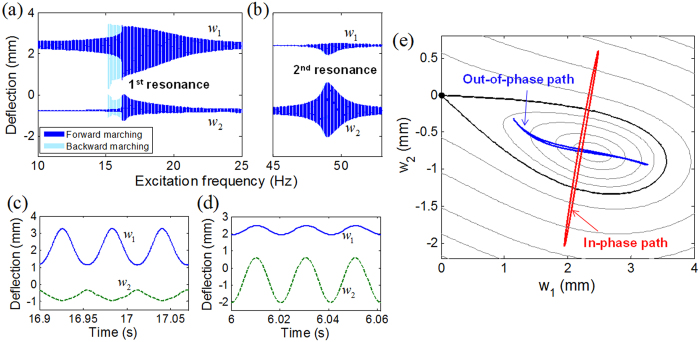
Out-of-phase and in-phase intrawell resonant motions obtained with a base acceleration of 2 m/s^2^. (**a,b**) The frequency marching responses obtained in the frequency regions for the 1^st^ and 2^nd^ primary resonances. (**c,d**) The stationary time responses of out of phase and in phase, respectively, evaluated at 17.5 Hz and 49 Hz, and (**e**) the associated paths intersecting the potential contour lines.

Figure 3 shows the 1^st^ and 2^nd^ intrawell resonant behaviors of deflections *w*_1_ and *w*_2_ obtained when the base acceleration is small (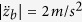). Without loss of generality, only intrawell motion within the right potential well is presented in Fig. 3 (also, all the result figures hereafter). As observed in Fig. 3(a,b), the vibration modes at the two resonances appear with the opposite trends of the amplitude ratios. The 1^st^ resonant mode tends to be of large *w*_1_ and small *w*_2_ (Fig. 3(a)), whereas the 2^nd^ resonant mode tends to be of small *w*_1_ and large *w*_2_ (Fig. 3(b)). This observation indicates that each of the two oscillators would possess its own frequency range in which it operates with a larger amplitude. The most remarkable feature is the phase difference between the 1^st^ and 2^nd^ resonant modes that make significant differences in the potential energy trajectories. From a series of simulations, deflections *w*_1_ and *w*_2_ are found to always oscillate out of phase in the frequency region of the 1^st^ resonance (Fig. 3(c)), but almost in phase in the region of the 2^nd^ resonance (Fig. 3(d)). For the out-of-phase mode, a strong magnetic repulsive force tends to form the potential energy barrier between the two magnets approaching each other, which prohibits the crossing-over of the oscillator motions. Thus, the 2-DOF BEH experiences strong stiffness softening at the 1^st^ intrawell resonance, and its frequency response tends to follow a typical hysteretic resonant curve (Fig. 3(a)). On the other hand, for the in-phase mode, the two magnets tend to oscillate simultaneously in the same direction and accordingly, typical bistable features such as the potential barrier and the softening effect are weakened significantly. The resulting resonant curve at the 2^nd^ resonance (Fig. 3(b)) is slightly bended without visible hysteresis and covers only a narrow frequency band. The above-mentioned behaviors of the out-of-phase and in-phase resonant modes can be obviously identified by tracing the associated potential energy trajectories. As illustrated in Fig. 3(e), the out-of-phase path intersects the contour lines of the potential energy along the direction of the skew-symmetric axis, being obstructed by the potential saddle where stiffness softening is maximized. The in-phase path is almost in the perpendicular direction to the skew-symmetric axis, so that it does not suffer from the potential barrier on its own path.

### Potential well escape phenomena under the out-of-phase and in-phase mode regimes

**Figure 4:**
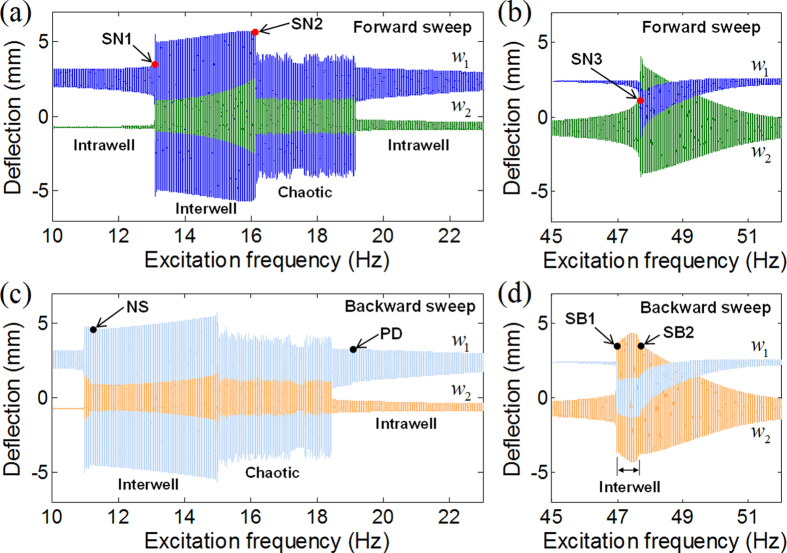
Interwell motions triggered by (the first column) the out-of-phase and (the second column) in-phase interwell resonances, when the base acceleration is 6 m/s^2^. In (**a,b**), points SN1–SN3 denote the saddle-node bifurcations where the jump-up or jump-down phenomenon of periodic oscillation can be observed. In (**c**), point PD means the period-doubling bifurcation, which leads to chaotic motion through a subsequent period-doubling cascade. Point NS is the Neimark–Sacker bifurcation, at which the periodic interwell motion is destabilized. In (**d**), points SB1 and SB2 indicate the subcritical and supercritical symmetry-breaking bifurcations, respectively.

When external excitation is strong, both the 1^st^ and 2^nd^ intrawell resonances can lead to the transition into the interwell motion, the so-called potential well escape phenomenon, but their processes are quite different. Figure 4 shows the interwell motions triggered by the out-of-phase and in-phase modes (the first and second columns, respectively) when the base acceleration is 6 m/s^2^. To demonstrate the difference in the transition processes, the forward and backward frequency-sweep responses (the first and second rows, respectively) are presented in this figure with several bifurcation points of periodic oscillations that can be identified based on the Floquet theory^36^ (refer to the Methods for the detailed procedures).

**Figure 5:**
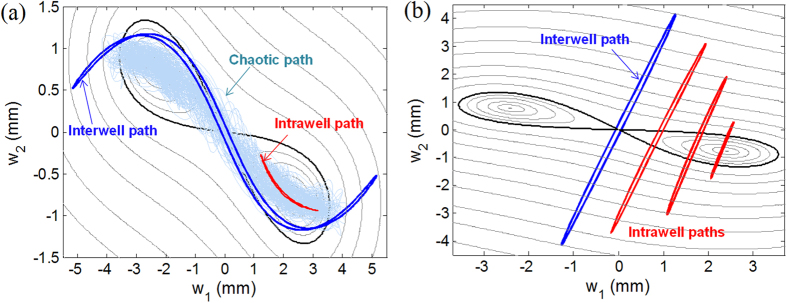
(**a**) Out-of-phase and (**b**) in-phase trajectories on the contour plot of the potential energy. In (**a**), the interwell, chaotic, and intrawell paths correspond to the stationary responses obtained at 14, 17, and 20 Hz, respectively, from Fig. 4(c). In (**b**), one interwell and three intrawell paths are obtained at 47.3, 48, 49, and 51 Hz, respectively, from Fig. 4(d).

The complicated bifurcation structure of the deflection response is observed particularly in the frequency region of the 1^st^ resonance. For the forward frequency-sweep response in Fig. 4(a), periodic interwell motion is initiated with a sudden jump-up phenomenon by the saddle-node bifurcation of the intrawell motion (designated by point SN1), then continues to grow with its amplitude (from SN1 to SN2), and finally jumps down through the chaotic region into the intrawell motion after passing the saddle-node bifurcation of the interwell motion (SN2). As for the backward frequency-sweep response in Fig. 4(c), the intrawell motion first experiences a period-doubling bifurcation (PD) and a subsequent period-doubling cascade that leads to chaotic motion. In this case, periodic interwell motion emerges after the chaotic motion disappears, and it persists until it becomes destabilized at the Neimark–Sacker bifurcation point (NS in Fig. 4(c)). In fact, such a bifurcation structure in the 1^st^ resonance is in accordance with those of existing 1-DOF BEHs^26^. This is because the out-of-phase trajectories of both chaotic and interwell motions always pass through the potential saddle in order to escape from one potential well into another, as can be seen in Fig. 5(a), and thus fundamental bistable natures are preserved without significant deterioration.

The bifurcation structure of the in-phase oscillation in the 2^nd^ resonance is simpler than that of the out-of-phase oscillation, as shown in Fig. 4(b,d), and it comprises one saddle-node and two symmetry-breaking bifurcations. For the forward frequency-sweep response in Fig. 4(b), interwell motion (if exists) would be abruptly activated by the saddle-node bifurcation of the intrawell motion (SN3). However, for the backward case in Fig. 4(d), the transition process from the intrawell to interwell motion is smooth and continuous. The asymmetric intrawell motion continuously gains its amplitude with the decrease in the excitation frequency, until it grows into symmetric interwell motion through a supercritical symmetry-breaking bifurcation (SB2). Then, the smoothly initiated interwell motion is terminated at its subcritical symmetry-breaking point (SB1 in Fig. 4(d)). Such bifurcation scenarios in the 2^nd^ resonance are not completely similar to those in the 1^st^ resonance, since the deflection responses remain almost in phase throughout the transition process. As depicted in Fig. 5(b), the intrawell oscillation of the in-phase mode does not seem to move across but gradually climbs during its growth. Then this intrawell motion disappears at the hilltop saddle, colliding with its skew-symmetric counterpart that has grown from the left potential well; instead, the interwell motion of the in-phase mode appears. This interwell motion does not reach deep inside either potential well, only staying around the hilltop saddle, which clearly indicates that the potential saddle does not act as a barrier.

**Figure 6:**
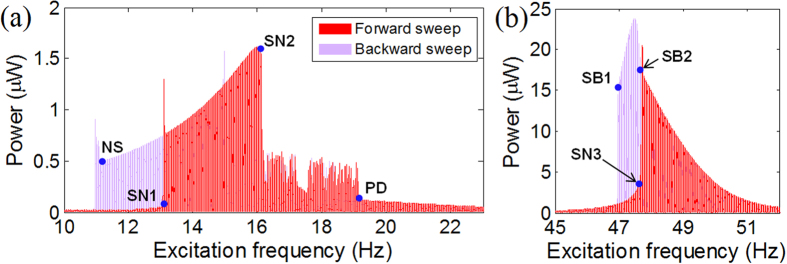
Frequency responses of the electrical output powers generated from the (**a**) left and (**b**) right bimorph beams, respectively, in the frequency regions of the (**a**) out-of-phase and (**b**) in-phase interwell motions.

The bifurcation structures discussed in Fig. 4 can consistently apply to the electrical output powers of the 2-DOF BEH. As shown in Fig. 6(a), the out-of-phase interwell motion produces an output power much higher than the chaotic or intrawell motion in a broad frequency band (between NS to SN2); whereas as in Fig. 6(b), the interwell motion of the in-phase mode is bounded in a narrower band (between SB1 and SB2). However, the intrawell motion of the in-phase mode can also generate a high output power, when it continuously grows towards point SB2. Thus, the operating frequency bandwidth of the 2-DOF BEH in the 2^nd^ resonance should be evaluated with such large-amplitude intrawell motion in addition to the interwell motion.

### Phase-dependent dynamic potential well

**Figure 7:**
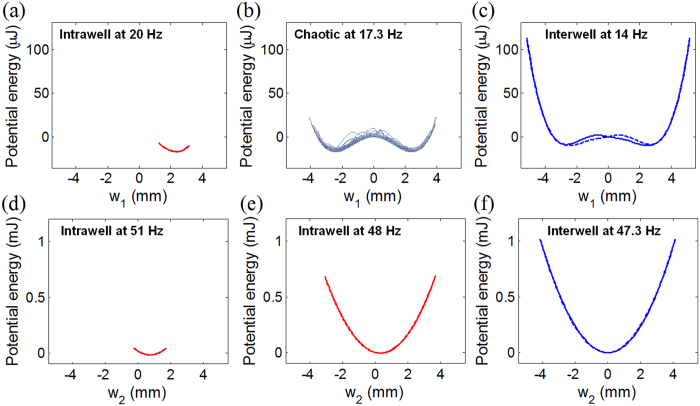
Dynamic potential well configurations for (the first row) the out-of-phase and (the second row) in-phase modes. The apparent dynamic potential wells illustrated in (**a–f**) are obtained at six different excitation frequencies used in Fig. 5. The solid and dashed lines denote the parts of positive and negative velocities in the stationary responses, respectively.

Figure 7 shows the potential well configurations that the motions of the out-of-phase and in-phase modes experience, obtained at six different excitation frequencies in Fig. 4. In this figure, for illustration, deflections *w*_1_ and *w*_2_ are chosen as the horizontal axes for the out-of-phase and in-phase modes, respectively. This result provides the most comprehensible demonstration on why the nonlinear dynamics of the 2-DOF BEH is not the same in the 1^st^ and 2^nd^ resonances. As shown in Fig. 7(a–c), the intrawell, chaotic, and interwell motions of the out-of-phase mode tend to form single-well (Fig. 7(a)), chaotic double-well (Fig. 7(b)), and symmetric double-well potential configurations (Fig. 7(c)), respectively. Thus, the underlying principle of the potential well escape phenomenon in the 1^st^ resonance follows the typical double-well dynamics of 1-DOF BEHs^26^. On the contrary, for the 2^nd^ resonance, the potential energy trajectories do not take any double-well configuration. As depicted in Fig. 7(d–f), only single-well potential configurations occur even for the interwell motion as well as the intrawell motion. Consequently, it can be concluded that the present 2-DOF BEH acts as a bistable system in the out-of-phase mode, but behaves as a monostable system in the in-phase mode.

The simulation results shown in Figs 2, 3, 4, 5, 6, 7 were obtained for the set of geometric dimensions given in Table 1. To further support our conclusion concerning the phase-dependent dynamics of the 2-DOF BEH system at primary resonances, additional simulation results (such as out-of-phase/in-phase trajectories and associated dynamic potential well configurations) were obtained for several different sets of geometric dimensions and are presented in Supplementary Figs S1 and S2.

## Conclusion

In this study, a series of theoretical analyses on a 2-DOF BEH was performed to investigate its complicated resonant behavior owing to the magnetic interaction between two movable magnets. The results revealed that the 2-DOF BEH possessed two distinct dynamic regimes: the out-of-phase mode in the 1^st^ resonance and the in-phase mode in the 2^nd^ resonance. Under the out-of-phase mode regime, the frequency response results of the 2-DOF BEH exhibited the classical double-well dynamics of bistable systems. Accordingly, the periodic out-of-phase interwell motion could generate a high output power in a broad frequency band bounded by the Neimark–Sacker and saddle-node bifurcations. However, under the in-phase mode regime, both intrawell and interwell motions belonged only to the single-well dynamics of monostable systems, even though the 2-DOF BEH must be statically bistable. In this case, the interwell motion branch originated from the supercritical or subcritical symmetry-breaking bifurcation bounded in a narrow frequency band, but the large-amplitude intrawell motion could additionally extend the operating frequency bandwidth for a high output power. As summarized above, this study has introduced the concept of the phase-dependent dynamic potential in the 2-DOF BEH for the first time, providing a clear physical insight into its nonlinear resonant behavior, including the bifurcation structures and the associated potential well escape phenomena. This concept is not limited to the present 2-DOF BEH, but can be extended and applied to all magnetically coupled multi-DOF BEHs, whose potential energies are functions of more than two displacement variables.

## Methods

For numerical simulations, second-order differential equations are transformed into an autonomous system of first-order differential equations and Eqs (1a) and (1b) can be written by using state vector 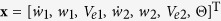 in a more convenient matrix-vector form:


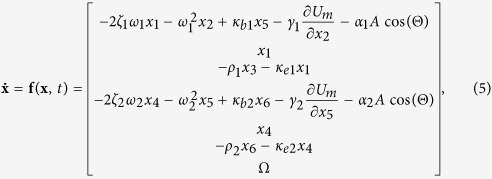


where Θ and Ω are the cyclic variable and the excitation angular frequency, respectively. The dynamic simulations are performed using the direct numerical integration for Eq. (5), and the stationary periodic solutions are obtained after a large number of forcing cycles (more than 10^3^ cycles), such that the steady-state condition is guaranteed. For all the numerical simulations, the following parameter values are used: *ζ*_*i*_ = (0.01, 0.01) for *i* = 1 and 2, respectively, *ω*_*i*_ = (1.73 × 10^2^, 3.30 × 10^2^), *κ*_*bi*_ = (−1.89 × 10^−3^, −3.13 × 10^−3^), *ρ*_*i*_ = (1.32 × 10^2^, 1.96 × 10^2^), *κ*_*ei*_ = (−3.34 × 10^3^, −7.49 × 10^3^), *α*_*i*_ = (1.17, 1.11), and *γ*_*i*_ = (7.47 × 10^2^, 8.21 × 10^2^). All of these parameter values were determined during the process of deriving the nonlinear oscillator model (specifically, the process of discretizing the governing field equations for the two bimorph beams). The geometric dimensions and material properties used to calculate the parameter values are listed in Table 1. For the purpose of demonstrating the phase-dependent dynamic potential, the geometric dimensions of the 2-DOF BEH system were chosen arbitrarily, within realistic ranges, based on the geometric configuration shown earlier in Fig. 1. The material properties of a stainless steel and a piezoelectric polyvinylidene fluoride were used for the metal substrates and piezo-layers of the bimorph beams. A resistance of 10 MΩ was used for external electrical loads *R*_1_ and *R*_2_.

The stability analysis for the stationary oscillation is performed based on the Floquet theory^36^. To this end, following monodromy matrix **M** of stationary oscillation **x**_*s*_(*t*) is constructed:


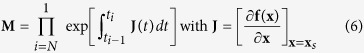


where **J** is the Jacobian matrix and *N* is the number of equally spaced subintervals (i.e., *t*_*i*_ − *t*_*i*−1_) during one period. Then Floquet multiplier *μ* can be obtained by solving the eigenvalue problem of **M**. The destabilization of **x**_*s*_ occurs when the largest magnitude of *μ* becomes 1, with certain bifurcation phenomena: saddle-node or symmetry-breaking bifurcation for *μ* = +1, period-doubling bifurcation for *μ* = −1, and Neimark–Sacker bifurcation for |*μ*| = 1 and 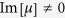. In this study, each type of bifurcations is identified in the frequency response results by examining the variations in the Floquet multipliers with the change in the excitation frequency.

## Additional Information

**How to cite this article**: Kim, P. *et al*. Phase-dependent dynamic potential of magnetically coupled two-degree-of-freedom bistable energy harvester. *Sci. Rep.* **6**, 34411; doi: 10.1038/srep34411 (2016).

## Supplementary Material

Supplementary Information

## Figures and Tables

**Table 1:** Geometric and material properties of the present system.

Parameter	Value
Substrates	
Length	70 mm (47 mm)^*^
Width	10 mm
Thickness	0.3 mm
Density	7850 kg/m^3^
Young’s modulus	200 GPa
Piezo-polymer layers	
Length	70 mm (47 mm)^*^
Width	10 mm
Thickness	0.052 mm
Density	1780 kg/m^3^
Young’s modulus	3 GPa
Piezo-strain constant	−23 pm/V
Permittivity	110 pF/m
Magnet	
Length	2 mm
Width	10 mm
Thickness	6 mm
Magnetization	9 × 10^5^ A/m

*The parameter value in parentheses is the length of the second bimorph beam.
