# Supplementary material for: Phase-dependent dynamic potential of magnetically coupled two-degree-of-freedom bistable energy harvester
Source: Sci Rep. 2016 Sep 28;6:34411. doi: 10.1038/srep34411 (PMC5039668; doi:10.1038/srep34411)
Supplement: Supplementary Information [file srep34411-s1.pdf]

Supplemental Material to:

Phase-dependent dynamic potential of magnetically coupled two-degree-of-freedom bistable energy harvester

Pilkee Kim[1], Minh Sang Nguyen[1,2], Ojin Kwon[1], Young-Jin Kim[1], and Yong-Jin Yoon[1,*]

[1]School of Mechanical and Aerospace Engineering, Nanyang Technological University, Singapore 639798, Singapore

[2]Energy Research Institute @ NTU, Interdisciplinary Graduate School, Nanyang Technological University, Singapore 639798, Singapore

[*]Corresponding Author: (Tel.) +65 6790 5033, (E-mail) yongjiny@ntu.edu.sg

As illustrated in Fig. 1(a) in the manuscript, it was assumed in this study that the two cantilever beam oscillators of the 2-DOF BEH system had the same geometric dimensions, except for their beam lengths. Without any loss of generality, because of the symmetric nature of the system, we considered only the case of the left beam shown in Fig. 1(a) being longer than the right beam. Supplementary Fig. S1 shows the out-of-phase/in-phase trajectories on the contour plot of the potential energy obtained for different lengths of the right beam, specifically, 57 mm, 52 mm, and 42 mm for Cases I, II, and III, respectively. All other geometric dimensions were the same as those listed in Table 1 in the manuscript. For each of the three cases, the equivalent parameter values for the nonlinear oscillator model (Eq. (5)) were calculated and are listed in Supplementary Table S1. Supplementary Fig. S2 shows the dynamic potential well configurations associated with the results shown in Supplementary Fig. S1. As Supplementary Figs. S1 and S2 show, all of the simulation results support the conclusion that the phase-dependent dynamic potential of the present 2-DOF BEH system leads to double-well dynamics for the out-of-phase mode at the 1st primary resonance but single-well dynamics for the in-phase mode at the 2nd primary resonance.

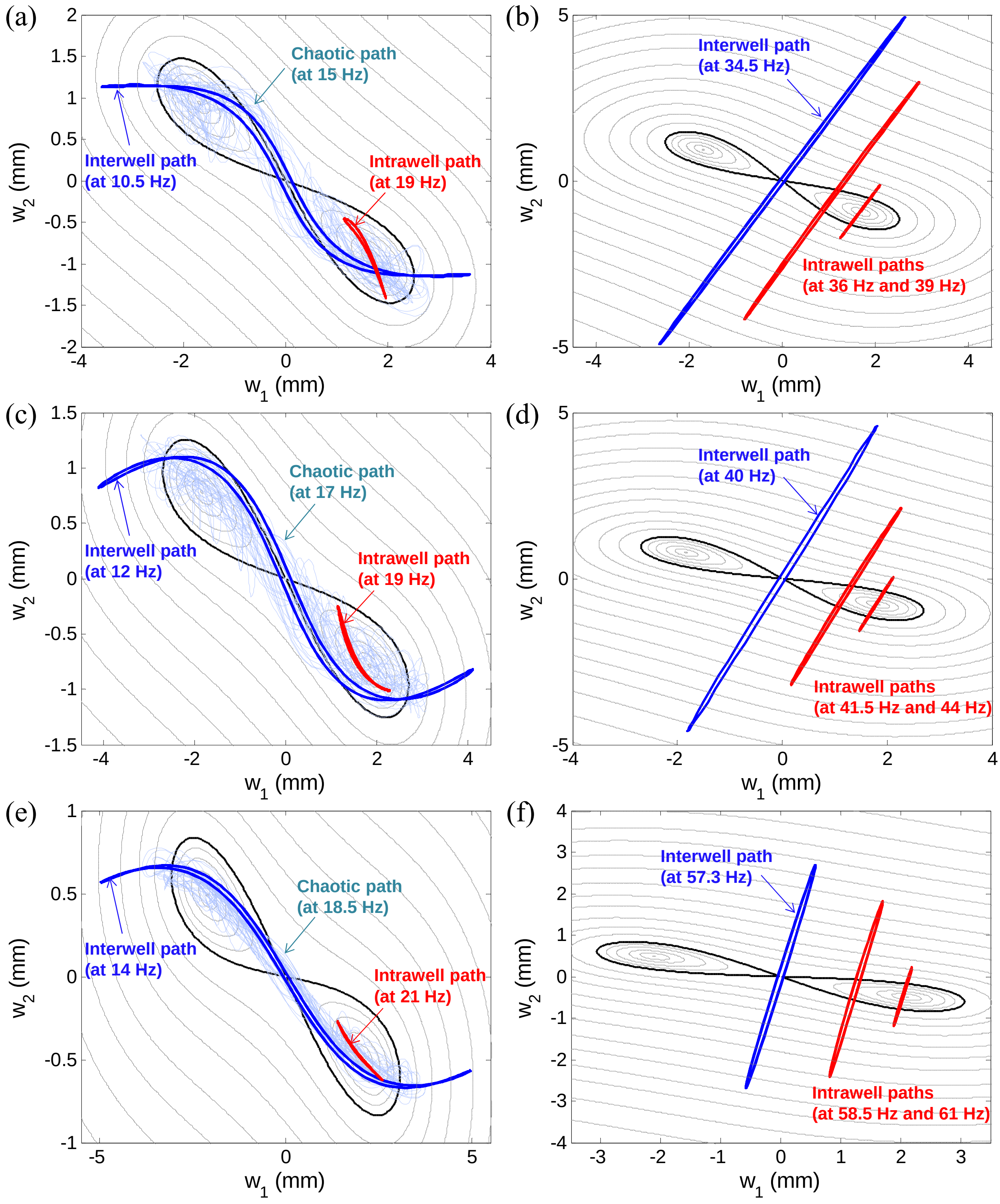

Supplementary Figure S1. (the first column) Out-of-phase and (the second column) in-phase trajectories on the contour plot of the potential energy. The potential energy trajectories are obtained for different lengths of the right beam, specifically, (Cases I) 57 mm, (Cases II) 52 mm, and (Cases III) 42 mm. The distance *d* is set to be 12.1 mm, 11.9 mm, and 11.5 mm for Cases I–III, respectively.

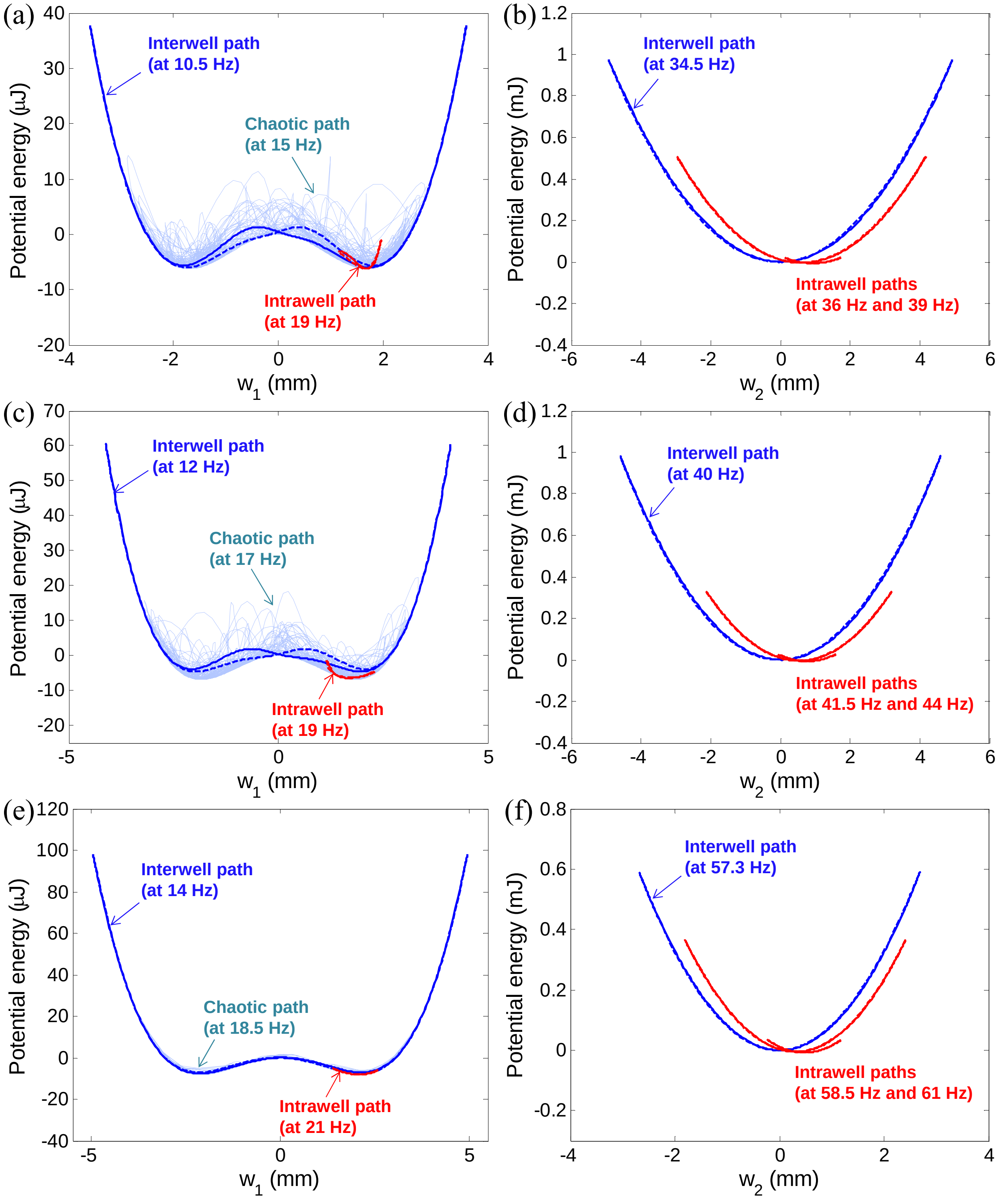

Supplementary Figure S2. Dynamic potential well configurations for (the first column) the out-of-phase and (the second column) in-phase modes. The six results presented in (a)–(f) are associated with those shown in Supplementary Fig. S1

Supplementary Table S1. System parameters of the nonlinear oscillator model for the Cases I–III

Parameter	Case I		Case II		Case III	
	i=1	i=2	i=1	i=2	i=1	i=2
ζ_i	0.01	0.01	0.01	0.01	0.01	0.01
ω_i	1.73×10^2	2.42×10^2	1.73×10^2	2.81×10^2	1.73×10^2	3.95×10^2
κ_{bi}	-1.89×10^{-3}	-2.47×10^{-3}	-1.89×10^{-3}	-2.77×10^{-3}	-1.89×10^{-3}	-3.58×10^{-3}
ρ_i	1.32×10^2	1.62×10^2	1.32×10^2	1.77×10^2	1.32×10^2	2.20×10^2
κ_{ei}	-3.34×10^3	-5.06×10^3	-3.34×10^3	-6.10×10^3	-3.34×10^3	-9.40×10^3
α_i	1.17	1.14	1.17	1.13	1.17	1.09
γ_i	7.47×10^2	7.88×10^2	7.47×10^2	8.04×10^2	7.47×10^2	8.36×10^2
